# Antibiotic Treatment Duration for Uncomplicated Monomicrobial Enterococcal Bloodstream Infection: A Multicenter Target Trial Emulation

**DOI:** 10.1093/cid/ciag387

**Published:** 2026-07-03

**Authors:** Hadar Mudrik-Zohar, Sean W X Ong, Steven Y C Tong, Tzipi Hornik-Lurie, Ayalla Sabbah, Orly Weinstein, Michal Chowers

**Affiliations:** Gray Faculty of Medical & Health Sciences, Tel-Aviv University, Tel-Aviv, Israel; Infectious Diseases Unit, Meir Medical Centre, Kfar-Sava, Israel; Department of Infectious Diseases, University of Melbourne at the Peter Doherty Institute for Infection and Immunity, Melbourne, Victoria, Australia; Victorian Infectious Diseases Service, Royal Melbourne Hospital at the Peter Doherty Institute for Infection and Immunity, Melbourne, Victoria, Australia; Department of Infectious Diseases, University of Melbourne at the Peter Doherty Institute for Infection and Immunity, Melbourne, Victoria, Australia; Victorian Infectious Diseases Service, Royal Melbourne Hospital at the Peter Doherty Institute for Infection and Immunity, Melbourne, Victoria, Australia; Institute of Health Policy, Management and Evaluation, University of Toronto, Toronto, Canada; Division of Infectious Diseases, Sunnybrook Health Sciences Centre, Toronto, Canada; Department of Infectious Diseases, University of Melbourne at the Peter Doherty Institute for Infection and Immunity, Melbourne, Victoria, Australia; Victorian Infectious Diseases Service, Royal Melbourne Hospital at the Peter Doherty Institute for Infection and Immunity, Melbourne, Victoria, Australia; Melbourne Centre for Clinical Trials, School of Population and Global Health, University of Melbourne, Melbourne, Australia; Department of Data Research at the Research Authority, Meir Medical Center, Kfar Saba, Israel; Department of Data Research at the Research Authority, Meir Medical Center, Kfar Saba, Israel; Deputy CEO and Head of Hospital's Division, Clalit Health Services, Israel; Gray Faculty of Medical & Health Sciences, Tel-Aviv University, Tel-Aviv, Israel; Infectious Diseases Unit, Meir Medical Centre, Kfar-Sava, Israel

**Keywords:** enterococcal bacteremia, uncomplicated bloodstream infection, antibiotic treatment duration, target trial emulation

## Abstract

**Background:**

Enterococcal bloodstream infection (BSI) is common with 30-day mortality rates approaching 25%–30%, yet evidence guiding optimal antibiotic treatment duration, particularly for patients with uncomplicated infection, is limited.

**Methods:**

We conducted a retrospective cohort study using a target trial emulation framework across 8 hospitals in Israel (2013–2023) of adults hospitalized with uncomplicated monomicrobial enterococcal BSI. Treatment strategies were defined as short (7 ± 2 days) versus long (14 ± 2 days) antibiotic therapy. A cloning-censoring-weighting framework with inverse probability of censoring weights was applied to account for immortal time bias and potential confounders. The primary outcome was 30-day all-cause mortality; secondary outcomes included 90-day all-cause mortality, recurrence, length of stay, readmission and *Clostridioides difficile* infection. Analyses were performed using pooled logistic regression and negative binomial regression models, with prespecified subgroup and sensitivity analyses.

**Results:**

Of 3450 patients with enterococcal bacteremia screened, 870 were eligible, and 485 received treatment durations consistent with the study treatment strategies (short n = 283, long n = 202). Mean age was 74 years (±15.0); 87/485 (18%) had pre-existing cardiac hardware, 164/485 (34%) a urinary source and 180/485 (37%) had nosocomial BSI. Adjusted analyses demonstrated no difference in 30-day mortality between short and long therapy (21.6% vs 21.3%; adjusted risk ratio 1.01, 95% CI 0.84 to 1.22). There was no evidence of differences across the secondary outcomes. Results were consistent across subgroup and sensitivity analyses.

**Conclusions:**

Short-course antibiotic treatment was associated with comparable clinical outcomes to long-course treatment in uncomplicated monomicrobial enterococcal BSI, supporting the use of short treatment durations in appropriately selected patients.

## Teaser

Short-course antibiotic therapy (7 ± 2 days) for uncomplicated enterococcal bloodstream infection demonstrated comparable outcomes to prolonged therapy (14 ± 2 days), including 30-day mortality, 90-day mortality and recurrent bacteremia within 90 days, supporting treatment shortening in appropriately selected patients.

**(See the Editorial Commentary by Parienti on pages e730–2.)**

*Enterococci* are gram-positive, facultative anaerobic cocci known to cause a wide range of infections in hospitalized patients, including urinary tract and intra-abdominal infections, central line-associated infections, primary bloodstream infections (BSIs) and infective endocarditis (IE) [1–3]. Due to their intrinsic resistance to multiple antibiotics and their ability to acquire and disseminate resistance genes through mobile genetic elements [4], *Enterococci* present significant treatment challenges.

Enterococcal BSI is common, often affects elderly and immunocompromised patients [1, 5], and is associated with substantial morbidity and 30-day mortality approaching 25%–30% [1, 3, 4, 6–12]. Population-based studies report an overall annual incidence of approximately 10 episodes per 100 000 persons [13–15], with rates reaching up to 76.6 cases per 100 000 population among adults aged >75 years [16]. In Israel, nationwide surveillance of hospital-onset BSIs identified *Enterococcus* spp. as the fourth most common cause of hospital-onset BSI, accounting for approximately 16% of cases [17]. BSIs caused by vancomycin-resistant *Enterococci* (VRE) are particularly concerning, with higher morbidity, mortality, and healthcare costs [12, 18–20]. In an Australian matched cohort, vanB VRE bacteremia was associated with an additional 4.9 hospital days and an adjusted increase in hospitalization costs of around $20 400 (USD) per episode compared with vancomycin-susceptible enterococcal bacteremia episodes [21]. Despite this substantial clinical and economic burden, specific treatment guidelines for enterococcal BSI are lacking, particularly in uncomplicated infections without evidence of IE or for infections caused by resistant strains.

While targeted antibiotic monotherapy (eg, ampicillin or vancomycin) is considered generally effective for uncomplicated enterococcal BSI, the optimal duration of treatment is a matter of debate. Clinicians often treat these patients for a range of 7–14 days [1, 20, 22, 23], yet high-quality evidence to guide whether shorter or longer durations in this range are preferred is scarce. This treatment dilemma is further complicated when patients at high-risk for complicated BSI (particularly IE in those with cardiac devices or a history of IE), present with apparently uncomplicated bacteremia, raising the question of whether long treatment may be warranted due to these underlying risk factors.

The BALANCE clinical trial showed that 7-day treatment duration is noninferior to 14 days for patients with uncomplicated BSI [24], however this was nonpathogen-specific, and clinical trials focused specifically on optimal duration for enterococcal BSI are still lacking. Here we employ a target trial emulation approach [25] to evaluate the impact of shorter versus longer antibiotic treatment durations on clinical outcomes in patients with uncomplicated enterococcal BSI over an 11-year period, across 8 medical centers in Israel.

## METHODS

### Study Design and Framework

We conducted a retrospective cohort study using the Clalit Health Services (CHS) electronic health record (EHR) database and applied a target trial emulation framework [25–29] to compare shorter versus longer antibiotic durations in adults with uncomplicated monomicrobial *E. faecalis* or *E. faecium* BSI across 8 CHS hospitals in Israel from January 2013 to October 2023 (details of participating hospitals are provided in Supplementary Table 1). The emulated target trial was designed as a pragmatic multicenter, open-label trial (Table 1).

**Table 1.** Target Trial and Emulation Designs

Protocol Component	Target Trial	Emulation Trial
**Eligibility criteria—**Inclusion criteria	Age ≥18 y at the time of admission. Hospitalized patients with enterococcal bacteremia based on at least 1 positive blood culture during their index hospitalization in one of the participating hospitals.	The same as for the target trial.
**Eligibility criteria—**Exclusion criteria	Patients diagnosed with endocarditis or other complications necessitating prolonged antibiotic treatment during their index hospitalization (including abscesses, bone and joint infections, cardiac or noncardiac devices related infections), until day 4 of hospitalization. Patients diagnosed with true polymicrobial bacteremia, defined as growth of *Enterococcus* spp. and other pathogens in the same blood culture, or within 48 h before or after the index culture. Cases in which bacterial isolated are classified as contaminants^a^ are not excluded. Prior inclusion in the study (only the first encounter of each patient was included). Appropriate antibiotic treatment was initiated more than 72 h after index blood culture collection (Supplementary Table 2 lists the empiric and definitive agents defined as active antienterococcal antibiotics). Patients who received less than 48 h of appropriate antibiotic treatment during hospitalization. Patients with blood cultures positive for *Enterococci* other than *E*. *faecalis* or *E*. *faecium* or unspecified species.	The same as for the target trial, except for criterion 1: Patients with endocarditis or other complications necessitating prolonged antibiotic treatment during their index hospitalization (including abscesses, bone and joint infections, cardiac or noncardiac device infections), were defined based on: Discharge ICD9/10 codes: An infectious diseases specialist reviewed the ICD codes to classify uncomplicated enterococcal bacteremia cases (see Supplementary Table 4). Prolonged treatment duration: Patients who received antibiotic treatment for more than 21 d were excluded, assuming their physicians classified them as having complicated enterococcal bacteremia. Additionally, patients who died or were discharged before day 4 from index blood culture collection were excluded as they could not be randomized on day 4, as well as those with missing antibiotic data.
**Treatment strategies**	Patients are randomly assigned on day 4 from index positive culture collection to either: Short treatment duration (7 ± 2 d): Continue treatment with appropriate antibiotics for a total duration of minimum of 5 d to a maximum of 9 d from index culture collection. Long treatment duration (14 ± 2 d): Continue appropriate antibiotic treatment for a total duration of a minimum of 12 d and a maximum of 16 d from index culture collection. Appropriate antibiotic treatment includes empiric and definitive antibiotic agents and is defined depending on susceptibility results (Supplementary Table 2). The selection of antibiotic agents, dosages and routes of administration are at the discretion of the treating physicians.	Treatment duration allocation was the same as for the target trial design.
**Treatment assignment**	Patients are randomized on day 4 from index positive culture collection in a 1:1 allocation ratio to a treatment strategy. Time zero is defined as the day of randomization. Investigators and patients are informed of the assigned treatment strategy on the day of randomization.	Randomization was emulated using the cloning, censoring and inverse probability of censoring weights (IPCW) approach. Patients were cloned at baseline to be included in both treatment groups and censored in line with their observed treatment. Differences in baseline characteristics and confounding by indication were addressed through the creation of IPCW.
**Follow-up**	Starts at time of randomization and ends at death or follow-up of 90 d after index culture collection, whichever occurs earliest.	Started from day 4 from index positive culture collection and ends at death or follow-up of 90 d after index culture collection, whichever occurs earliest.
**Outcomes**	Primary outcome: all-cause mortality within 30 d of the index positive enterococcal blood culture collection. Secondary Outcomes: All-cause mortality within 90 d of index positive enterococcal blood culture collection. Microbiological relapse of enterococcal bacteremia, defined as a positive blood culture for the same enterococcal species occurring more than 21 d from index culture and after completion of antibiotic treatment, within 90 d of index positive enterococcal blood culture during the index or recurrent readmissions. Total length of hospital stay. Length of hospital stay post index positive culture. Recurrent admissions from any reason within 90 d of the index positive enterococcal blood culture. *Clostridioides difficile* infection within 90 d of index positive enterococcal blood culture, defined as a positive glutamate dehydrogenase (GDH) antigen and toxin enzyme immunoassay (EIA), or in the case of discrepant results, a positive nucleic acid amplification test (NAAT) for the toxin in stool during the 90-d follow-up after the positive index culture.	Same as for the target trial.
**Causal contrasts (Treatment effects)**	Treatment effect in the intention to treat (ITT) population and the per-protocol (PP) population.	Treatment effect in the per protocol population.
**Analysis plan**	For ITT and PP analyses: logistic regression is used to calculate adjusted risk ratios and risk differences of all cause 30-d mortality, comparing patients who received short versus long antibiotic treatment.	PP effects were estimated using IPCW-adjusted pooled logistic regression models to compare adjusted risk ratios and risk differences for all-cause 30-d mortality associated with each treatment strategy, applying the cloning-censoring-weighting approach. To account for confounding by indication and selection biases, an IPCW were estimated from Cox models for the probability of remaining uncensored within each treatment arm, incorporating baseline patient variables and hospital as a random effect. Sensitivity analyses included repeating the model using treatment duration cut-off of 8 d (based on the observed median of actual distribution of treatment duration), and rerunning models without random effects for hospital sites.

Abbreviation: ICD—International Classification of Diseases.

^a^Based on the centers for disease control and prevention (CDC)'s national healthcare safety network (NHSN) organisms list. Available from: https://www.cdc.gov/nhsn/pdfs/validation/2019/2019-NHSN-Organisms-List-Validation.xlsx.

The primary outcome was 30-day all-cause mortality; measured from the date of the first positive blood culture (“index culture”). Secondary outcomes included 90-day all-cause mortality, microbiological recurrence, readmission, *Clostridioides difficile* infection (CDI) and hospital length of stay (Table 1).

Time zero for the emulated trial was prespecified as day 4 after the collection of the index culture, reflecting the typical timeframe required to review diagnostic work-up and reasonably exclude complicated infection in real-life clinical practice. To minimize immortal time bias, we excluded patients who died or were not under observation before day 4, as these individuals could not have been eligible for assignment to either treatment strategy at time zero.

### Exposure Definition

The exposure of interest was the duration of appropriate antibiotic therapy, defined as receipt of an agent active against the isolated *Enterococcus* species based on in-vitro susceptibility testing (further details are provided in the supplementary Clinical definitions and assumptions section and Supplementary Table 2). Treatment strategies were defined as short (7 ± 2 days) versus long (14 ± 2 days) courses of total appropriate antienterococcal therapy, counted from the collection date of the index culture. Duration encompassed both inpatient therapy and postdischarge prescriptions.

### Data Sources

Patients were identified through EHRs from 8 hospitals in the CHS network, Israel's largest healthcare organization, which covers approximately 50% of the Israeli population. The cohort represents a broad healthcare landscape, including 2 tertiary referral centers, 5 secondary hospitals, and 1 peripheral regional hospital, comprising a total of 4898 admission beds (Supplementary Table 1). Clalit maintains an integrated inpatient-outpatient EHR ecosystem including linked pharmacy dispensing data, enabling longitudinal patient-level follow-up across care settings. Data were extracted from CHS using the Clalit Research Data sharing platform powered by MDClone [30]. Extraction was performed by the Sharon-Shomron research team at Meir medical center, which has authorized access to Clalit's datasets. Demographic, clinical, microbiological and antibiotic prescribing and administration data (including postdischarge dispensing records) were collected, de-identified, and transferred to Clalit's secure virtualization platform. Dataset cleaning and construction were performed by the Gertner Institute of Health Policy and Epidemiology team.

### Study Population

Adults hospitalized during the study period with at least 1 positive enterococcal blood culture were screened. Uncomplicated monomicrobial BSI was defined as a bloodstream infection caused solely by *E. faecalis* or *E. faecium*, without evidence of IE, abscess, bone or joint infection, device-related infection, or other deep-seated focus. Classification of uncomplicated versus complicated infection, as well as the source of BSI, were determined by an infectious diseases specialist based on review of patients’ discharge International Classification of Diseases (ICD) codes (Supplementary Table 4). Patients were excluded from the target trial emulation analysis if they had discharge diagnoses indicating a complicated BSI, polymicrobial bacteremia, hospitalization of 4 days or less (required for trial emulation), or received appropriate antibiotic treatment durations shorter than 2 days or longer than 21 days, the latter of which was considered suggestive of a clinically complicated infection. Inclusion and exclusion criteria are detailed in Table 1; clinical definitions and assumptions are described in the Supplementary materials.

### Statistical Analysis

The primary analysis estimated the treatment effect in the per-protocol population by comparing short versus long therapy on clinical outcomes. Treatment assignment was emulated using cloning, censoring, and inverse probability of censoring weighting (IPCW) approach to account for deviation from the assigned treatment strategies and informative censoring [26–29] (Figure 1, Table 1 and Supplementary methods section). IPCW-weighted pooled logistic regression models were used and adjusted risk ratios and risk differences were reported. Nonparametric bootstrapping with 500 samples (with replacement) was used for estimation of 95% confidence intervals (CI). For continuous secondary outcomes IPCW-weighted negative binomial regression models were used to estimate adjusted rate ratios. Covariate balance before and after IPCW was assessed using standardized mean differences (SMDs), with SMD <0.10 considered adequate balance. Balance was visualized using a Love plot [31, 32].

**Figure 1.**
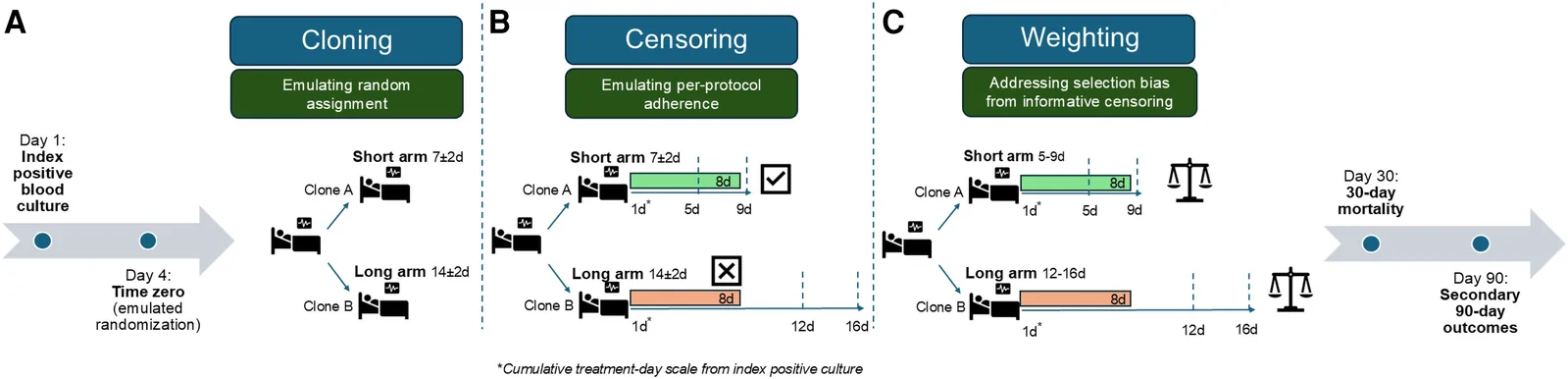
Overview of cloning, censoring, and weighting framework: (*A*) Cloning: Each eligible patient with uncomplicated enterococcal bloodstream infection was cloned into both treatment strategies: short (7 ± 2 d) and long (14 ± 2 d) to emulate random assignments at day 4 (time zero). (*B)* Censoring: Patients were censored when their observed treatment duration deviated from the assigned strategy. In the example shown, a patient treated for 8 d remains uncensored in the short-arm clone but is censored in the long-arm clone on day 9, the point at which he deviates from his assigned strategy. (*C)* Weighting: Inverse probability of censoring weights (IPCW) were used to adjust for informative censoring. Patients with a lower probability of remaining uncensored (ie, more likely to be censored) were upweighted, whereas those with a higher probability of remaining uncensored were downweighted, ensuring that the weighted sample represented the full eligible population. Stabilized weights were then applied to the outcome models to estimate treatment effects under the assumption of noninformative censoring after weighting. *Day markings (eg, 1d, 5d, 9d, 12d, 16d) in the Censoring and Weighting (B and C) represent cumulative treatment days measured from the index positive blood culture, not days from randomization.*

Prespecified subgroup analyses explored potential heterogeneity of treatment effect according to species (*E. faecalis* vs *E. faecium*), source of BSI (urinary tract, intra-abdominal, vascular catheter-related, gynecological or skin/soft tissue, vs unspecified source), empiric antienterococcal therapy (receipt of an appropriate empiric agent with activity against *Enterococci* from the time of index culture collection vs no such coverage), presence versus absence of pre-existing cardiac device, and site of acquisition (community- vs hospital-onset).

We conducted 2 sensitivity analyses to assess the robustness of the primary findings to alternative exposure definitions and modeling assumptions: (a) fitting models without random effects for hospital sites, and (b) redefining treatment strategies using an 8-day cutoff (≤8 days vs >8 days), corresponding to the median of the observed distribution of actual treatment durations administered.

The statistical analyses were performed using R version 4.4.2 (foundation for Statistical Computing, Vienna, Austria) with RStudio 2022.07.1 build 554 (Posit Software, Boston MA) [33]. The following R packages were used: tableone, tidyverse, survival, cobalt, boot, MASS, and epitools.

### Ethics

The study was approved by the CHS ethics committee in accordance with the Helsinki Declaration (MMC233-23). Informed consent was waived as the study used only fully de-identified data extracted from the Clalit integrated EHR records dataset. According to local privacy regulations and health data policies, secondary analysis of anonymized data does not require individual consent.

## RESULTS

### Cohort Characteristics

Between January 2013 and October 2023, 3450 adult patients with enterococcal bacteremia were screened. 870 met eligibility criteria for uncomplicated monomicrobial BSI. Of these, 485 received antibiotic treatment durations consistent with the predefined strategies: 283 (58%) in the short-course group (7 ± 2 days) and 202 (42%) in the long-course group (14 ± 2 days) (Figure 2). Among the 485 included patients, the mean age was 74 years (SD 15.0) and 175 (36%) were female (Table 2). Overall, 243 (50%) had a high comorbidity burden as indicated by modified Charlson comorbidity score ≥5 (excluding age to account for the elderly-skewed cohort) and 87 (18%) had a pre-existing cardiac device (prosthetic heart valves or cardiac implantable electronic devices). *E. faecalis* accounted for 404 (83%) cases, while VRE were uncommon, isolated in 9 patients (2%). The urinary tract was the most frequent source of infection (164 patients; 34%), followed by intra-abdominal sources (67 patients; 14%); and 180 (37%) had hospital-acquired episodes. IPCW achieved satisfactory covariate balance across treatment strategies, with all SMD <0.1 following weighting (Supplementary Figure 1).

**Figure 2.**
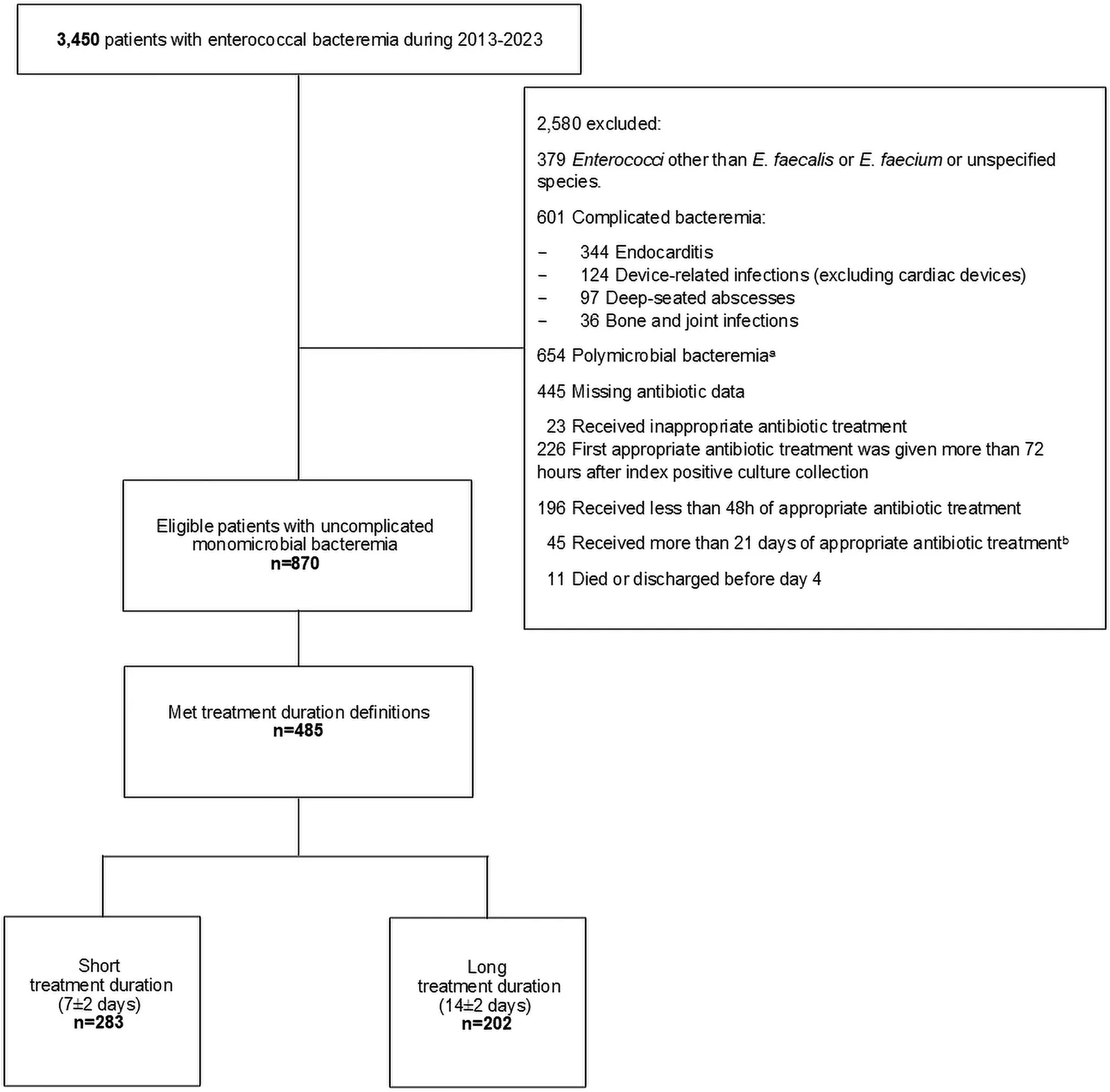
Study flowchart and cohort assembly. ^a^ Defined as patients with growth of *E. faecalis* or *E. faecium* and at least one other pathogen in the same blood culture, or within 48 h before or after the index culture. Cases in which bacteria classified as contaminants were isolated, with no true other pathogens present, were not excluded. ^b^ Extended antibiotic courses beyond 21 d were interpreted as indicative of a clinically complicated infection.

**Table 2.** Baseline Patient Characteristics

Variable	Overall n = 485	Short Treatment 7+/−2 D n = 283	Long Treatment 14+/−2 D n = 202	SMD
**Demographics**				
Age	74.5 (15.0)	74.9 (15.3)	74.1 (14.6)	0.053
Female sex	175 (36.1)	110 (38.9)	65 (32.2)	0.140
**Comorbidities**				
Median BMI (IQR)	27 (23.0–31.0)	27 (24.0–31.3)	27 (24.0–31.0)	0.033
CCI (age excluded)	…	…	…	0.098
Mild (1–2)	101 (20.8)	55 (19.4)	46 (22.8)	…
Moderate (3–4)	141 (29.1)	81 (28.6)	60 (29.7)	…
Severe (≥5)	243 (50.1)	147 (51.9)	96 (47.5)	…
Diabetes mellitus	247 (50.9)	151 (53.4)	96 (47.5)	0.117
Chronic kidney disease	198 (40.8)	123 (43.5)	75 (37.1)	0.129
Congestive heart failure	163 (33.6)	100 (35.3)	63 (31.2)	0.088
Ischemic heart disease	107 (22.1)	60 (21.2)	47 (23.3)	0.050
Cardiac device	87 (17.9)	47 (16.6)	40 (19.8)	0.083
Prosthetic heart valve	50 (10.3)	25 (8.8)	25 (12.4)	0.115
CIED	46 (9.5)	25 (8.8)	21 (10.4)	0.053
Cerebrovascular accident	195 (40.2)	110 (38.9)	85 (42.1)	0.065
Pulmonary disease	150 (30.9)	91 (32.2)	59 (29.2)	0.064
Solid malignancies	161 (33.2)	99 (35.0)	62 (30.7)	0.091
Hematologic malignancies	45 (9.3)	25 (8.8)	20 (9.9)	0.037
Dementia	110 (22.7)	68 (24.0)	42 (20.8)	0.078
Liver diseases	65 (13.4)	39 (13.8)	26 (12.9)	0.027
Peripheral vascular disease	77 (15.9)	36 (12.7)	41 (20.3)	0.205
Rheumatic diseases	36 (7.4)	17 (6.0)	19 (9.4)	0.128
**Hospitalization details**				
Year of infection	…	…	…	0.112
2013–2016	95 (19.6)	56 (19.8)	39 (19.3)	…
2017–2020	223 (46.0)	124 (43.8)	99 (49.0)	…
2021–2023	167 (34.4)	103 (36.4)	64 (31.7)	…
Hospital	…	…	…	0.148
Tertiary	220 (45.4)	137 (48.4)	83 (41.1)	…
Secondary	263 (54.2)	145 (51.2)	118 (58.4)	…
Regional	2 (0.4)	1 (0.4)	1 (0.5)	…
Admission ward	…	…	…	0.124
Internal medicine	354 (73.0)	206 (72.8)	148 (73.3)	…
Surgery	107 (22.1)	62 (21.9)	45 (22.3)	…
Gynecology	12 (2.5)	9 (3.2)	3 (1.5)	…
Intensive care unit	12 (2.5)	6 (2.1)	6 (3.0)	…
**Infection severity**				
Mechanical ventilation at baseline	16 (3.3)	11 (3.9)	5 (2.5)	0.081
Vasopressors use at baseline	43 (8.9)	28 (9.9)	15 (7.4)	0.088
Median creatinine (gr/dL) (IQR)	1.2 (0.9–1.9)	1.3 (0.9–2.1)	1.2 (0.8–1.8)	0.071
**Bacteremia characteristics**				
Source of Infection	…	…	…	0.237
Urinary tract	164 (33.8)	98 (34.6)	66 (32.7)	…
Intra-abdominal	67 (13.8)	37 (13.1)	30 (14.9)	…
Vascular catheter-related	6 (1.2)	2 (0.7)	4 (2.0)	…
Gynecological	5 (1.0)	5 (1.8)	0 (0.0)	…
Skin/soft tissue	4 (0.8)	3 (1.1)	1 (0.5)	…
Unspecified	239 (49.3)	138 (48.8)	101 (50.0)	…
Hospital-onset bacteremia	180 (37.1)	109 (38.5)	71 (35.1)	…
Median days from collection to result (IQR)	0.6 (0.5–1.3)	0.6 (0.5–1.3)	0.7 (0.5–1.4)	0.177
Median days of bacteremia (IQR)	1.0 (1.0–1.0)	1.0 (1.0–1.0)	1.0 (1.0–2.0)	0.082
Total positive blood culture bottles^a^	…	…	…	0.247
1	232 (47.8)	147 (51.9)	85 (42.1)	…
2	124 (25.6)	73 (25.8)	51 (25.2)	…
3–5	100 (20.6)	49 (17.3)	51 (25.2)	…
>5	29 (6.0)	14 (4.9)	15 (7.4)	…
*Enterococci* Species	…	…	…	0.006
*E. faecalis*	404 (83.3)	236 (83.4)	168 (83.2)	…
*E. faecium*	81 (16.7)	47 (16.6)	34 (16.8)	…
Ampicillin susceptibility (%)	…	…	…	0.111
Susceptible	408 (84.1)	241 (85.2)	167 (82.7)	…
Nonsusceptible	63 (13.0)	36 (12.7)	27 (13.4)	…
Not performed or missing	14 (2.9)	6 (2.1)	8 (4.0)	…
Vancomycin-resistant *Enterococci*	9 (1.9)	7 (2.5)	2 (1.0)	0.114
**Antibiotic treatment**				
Appropriate empiric antibiotics	94 (19.4)	60 (21.2)	34 (16.8)	0.112
Median days from culture collection to appropriate antibiotic treatment (IQR)	1.6 (0.8–2.4)	1.6 (0.8–2.5)	1.6 (0.8–2.4)	0.048
Combination therapy^b^	191 (39.4)	109 (38.5)	82 (40.6)	0.043
**Imaging**				
Echocardiography^c^	…	…	…	0.535
Not performed	230 (47.4)	161 (56.9)	69 (34.2)	…
Transthoracic	220 (45.4)	113 (39.9)	107 (53.0)	…
Transesophageal	35 (7.2)	9 (3.2)	26 (12.9)	…
PET/CT scan	18 (3.7)	9 (3.2)	9 (4.5)	0.067

Data are mean (SD) or n (%), except for variables explicitly reported as median (IQR).

Abbreviations: BMI, body mass index; CCS, Charlson Comorbidity Score (calculated without age adjustment to account for elderly skewed cohort); CIED, cardiac implantable electronic devise; PET/CT, positron emission tomography/computed tomography; SMD, standardized mean difference.

^a^Counts may include multiple cultures drawn on the same day.

^b^Combination therapy defined as administration of ceftriaxone or gentamicin in conjunction with antibiotics with direct enterococcal activity.

^c^Patients who underwent both transthoracic echocardiography (TTE) and transesophageal echocardiography (TEE) were classified to the TEE group.

### Primary Analysis

In unadjusted crude analyses that did not account for baseline differences or immortal-time bias, 30-day mortality was 31.8% versus 9.9% in the short and long treatment duration groups. When patients who died within the first 14 days were excluded, 30-day mortality was nearly identical between groups (n = 409, 8.1% vs 8.5%) (Supplementary Figures 2 and 3).

After applying the target trial emulation framework, IPCW-weighted analyses showed similar 30-day all-cause mortality between short and long treatment groups (adjusted absolute risk 21.6% vs 21.3%; adjusted risk difference 0.3%; 95% CI, −3.7% to 4.2%; adjusted risk ratio [aRR] 1.01; 95% CI, 0.84 to 1.22) (Table 3 and Figure 3). No significant differences were observed for 90-day all-cause mortality (37.2% vs 38.1%; aRR 0.98; 95% CI, 0.85 to 1.11), microbiological recurrence (aRR 0.95; 95% CI, 0.60 to 1.54), CDI (aRR 0.97; 95% CI, 0.38 to 2.03), or all-cause readmissions (aRR 1.00; 95% CI, 0.90 to 1.14) (Table 3). Continuous outcomes were likewise comparable, including total length of hospital stay (mean 24.7 vs 24.9 days; risk ratio 1.01; 95% CI, 0.93 to 1.11), and length of stay measured from index culture (mean 17.2 vs 17.9 days; risk ratio 1.04; 95% CI, 0.97 to 1.13).

**Figure 3.**
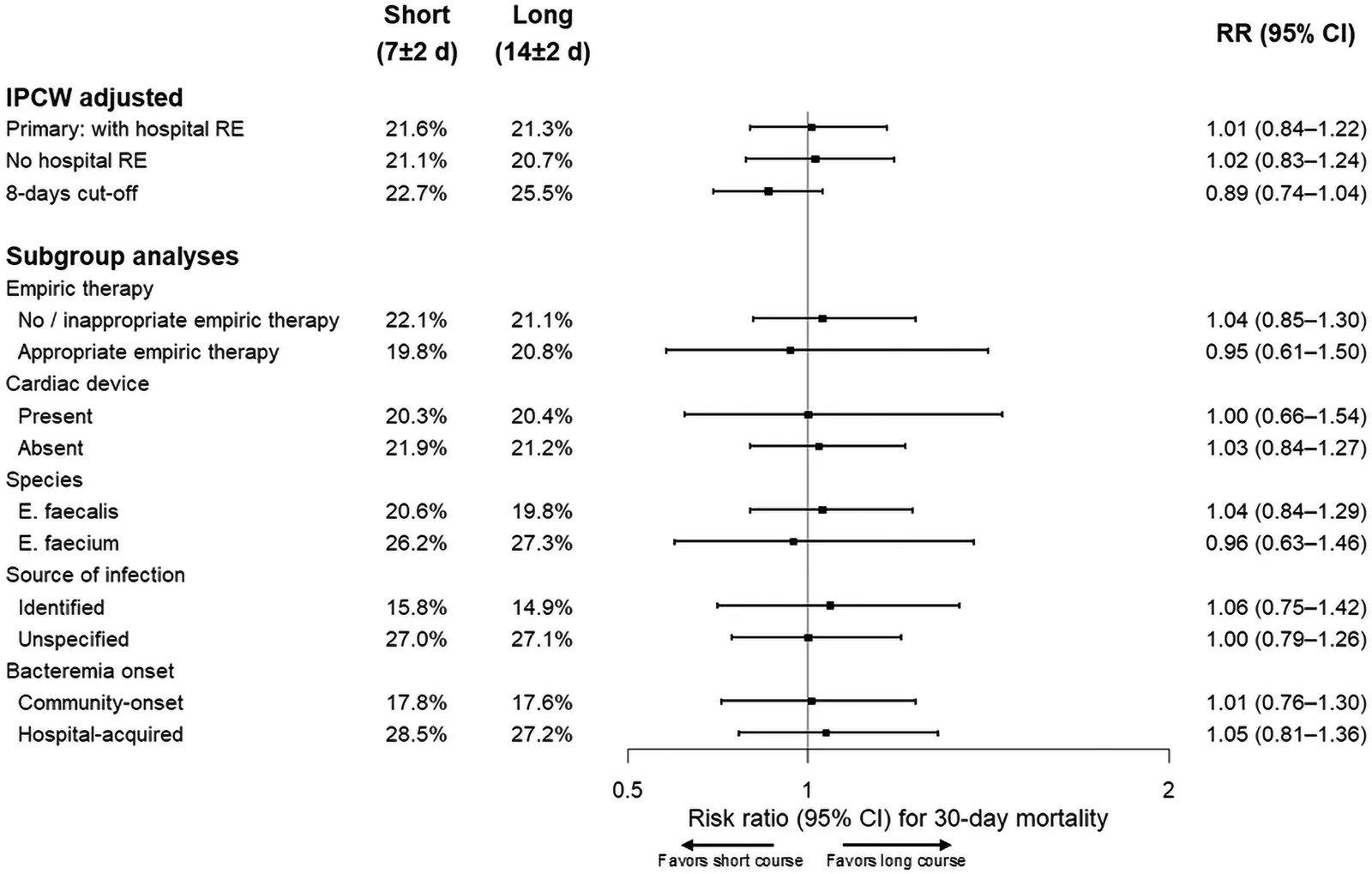
IPCW-weighted 30-d mortality analyses. The top panel shows the primary IPCW-weighted analysis incorporating a hospital-level random effect (RE), followed by a sensitivity analysis without the random effect and an analysis using an 8-d cutoff for treatment duration. The lower panel presents prespecified IPCW-weighted subgroup analyses. The right-hand column displays the risk ratio (RR) and corresponding 95% CI. IPCW were estimated from Cox proportional hazards models including age, sex, admission department, cardiac device status, Charlson Comorbidity Index (minus age), infection source, hospital-onset bacteremia, enterococcal species, vancomycin resistance status, vasopressor use, creatinine level, and year of admission, with hospital site included as a random effect.

**Table 3.** IPCW-weighted Adjusted Absolute Risks and Treatment Effect Estimates for Primary and Secondary Outcomes

Outcome	Absolute Risk Short Treatment 7 ± 2 D	Absolute Risk Long Treatment 14 ± 2 D	Risk Difference (95% CI)	Risk Ratio (95% CI)
**Primary outcome**				
30-d all-cause mortality	0.215 (0.187–0.243)	0.212 (0.185–0.243)	0.003 (−0.037–0.042)	1.014 (0.841–1.215)
**Secondary outcomes**				
90-d all-cause mortality	0.372 (0.340–0.404)	0.381 (0.348–0.421)	−0.009 (−0.064–0.041)	0.977 (0.847–1.112)
Microbiological recurrence	0.046 (0.032–0.060)	0.048 (0.033–0.066)	−0.002 (−0.023–0.020)	0.954 (0.602–1.542)
*Clostridioides difficile* infection	0.019 (0.009–0.029)	0.020 (0.010–0.031)	−0.001 (−0.016–0.013)	0.970 (0.384–2.033)
Recurrent admissions	0.451 (0.413–0.485)	0.449 (0.414–0.486)	0.002 (−0.049–0.058)	1.004 (0.896–1.137)

Absolute risks reflect inverse probability of censoring weighted (IPCW)—marginal estimates from pooled logistic regression models. Adjusted risk differences were calculated as short minus long, and adjusted risk ratios >1 indicate higher risk with short-course therapy. Stabilized IPCW were derived from Cox proportional hazards models including age, sex, admission department, cardiac device status, Charlson Comorbidity Index (excluding age), source of infection, hospital-onset bacteremia, enterococcal species, vancomycin resistance status, vasopressor use, baseline creatinine, and year of admission, with hospital site modeled as a random effect. Secondary outcomes were assessed through 90 d after the index positive culture. .

### Subgroup Analyses

Prespecified subgroup analyses were conducted for the primary outcome. No significant differences in 30-day mortality were observed across subgroups defined by empiric antienterococcal therapy (administered vs not), presence of cardiac devices (present vs absent), *Enterococcus* species (*E. faecalis* vs *E. faecium),* infection source (identified vs unspecified) or site of onset (hospital vs community) (Figure 3).

Given the potential association between intracardiac devices and complicated infection or recurrence, we performed an exploratory analysis of 90-day microbiological recurrence by cardiac device status. Among patients with cardiac devices, recurrence occurred in 9.6% of the short-therapy group versus 9.1% of the long-therapy group (aRR 1.06; 95% CI, 0.50–2.08; Supplementary Table 5). Among patients without cardiac devices, recurrence rates were 3.7% versus 4.2%, respectively (aRR 0.88; 95% CI, 0.45–1.57). These findings did not suggest differential effects of treatment duration on recurrence according to device status.

### Sensitivity Analyses

Sensitivity analyses were consistent with the primary findings. First, estimates were similar when IPCW were generated without including hospital as a random effect (30-day mortality; aRR 1.02; 95% CI, 0.83 to 1.24) (Figure 3). Second, in the full cohort (n = 870), the crude median treatment duration was 8.6 days, reflecting the observed real-world prescribing distribution. To further assess robustness, we performed an additional analysis in this full cohort without restricting patients to the predefined treatment duration strategies of the primary target trial emulation. Instead, treatment duration was categorized as ≤8 days (n = 390) versus >8 days (n = 480) (Supplementary Tables 6 and Figure 4). After applying the cloning-censoring-weighting framework with IPCW, 30-day mortality was 22.7% in the ≤8 days group compared with 25.5% in the >8 days group (aRR 0.89; 95% CI, 0.74 to 1.04) (Supplementary Table 7). Findings were similar for 90-day mortality (38.7% vs 40.6%, aRR 0.95; 95% CI, 0.85 to 1.07) and for all other secondary outcomes (Supplementary Tables 7).

## DISCUSSION

In this large multicenter target trial emulation study, short-course antibiotic therapy (7 ± 2 days) resulted in outcomes comparable to long therapy (14 ± 2 days) in adults with uncomplicated monomicrobial enterococcal BSI. Using a cloning-censoring-weighting framework with IPCW to mitigate immortal time and selection biases, 30-day all-cause mortality was similar between strategies, with concordant findings across secondary outcomes, prespecified subgroups, and sensitivity analyses. These results support the effectiveness and safety of shorter treatment in appropriately selected patients.

The optimal duration of antibiotic therapy for enterococcal BSI has not been well studied, and current practice is largely based on expert opinion rather than high-quality evidence. Guidelines and reviews suggest 7–14 days for “uncomplicated” bacteremia, reflecting wide uncertainty [5, 6]. Clinical severity definitions are also inconsistent: although no standardized criteria exist, uncomplicated enterococcal BSI is generally short-lived, linked to a clear and controlled source, clears promptly with active therapy, and with no evidence of IE or deep foci; while “complicated” BSI is typically persistent and associated with IE, abscesses, bone and joint infections, device-related infections, or other metastatic foci of infection [5, 34]. The difficulty in confidently classifying cases as “uncomplicated” complicates antibiotic-duration decisions and discourage clinicians from shortening therapy.

There is robust randomized evidence establishing the safety of short courses in Gram-negative BSIs, including RCTs and meta-analyses showing 7 days is sufficient in uncomplicated BSIs [35, 36]. In contrast, the evidence for Gram-positive BSIs, including enterococcal BSI is limited, with treatment duration recommendations largely extrapolated from expert opinion or nonpathogen-specific studies [24]. Hence, practice patterns for uncomplicated enterococcal bacteremia remain highly variable. An international survey highlighted substantial inconsistency in recommended treatment duration for enterococcal BSI among infectious diseases specialists, reflecting the absence of definitive trial data and uncertainty in clinical decision-making [37]. The recently published BALANCE trial, the largest randomized trial to date on treatment duration in BSI of diverse causative agents, demonstrated noninferiority of 7 days versus 14 days of therapy for 90-day mortality, potentially even for bacteremia caused by *Streptococci* and *Enterococci* [24]. The BALANCE trial enrolled 3608 patients with uncomplicated bacteremia, of whom 250 had enterococcal bacteremia. Species-specific subgroup results were not reported, limiting direct inference for *Enterococci*. Specific to *Enterococci*, a multicenter retrospective cohort by Bahrs et al reported similar 30-day mortality for short (≤9 days) versus long (≥10 days) courses in 219 uncomplicated vancomycin-resistant enterococcal bacteremia cases (19.2% vs 22%, adjusted odds ratio 1.15; 95% CI, 0.52 to 2.56) [38]. Additionally, an observational study in uncomplicated enterococcal catheter-related infections, similarly failed to show clear benefit from long therapy [39]. Our findings, aligning with the results of these previous studies, suggest that routine 14-day courses are often unnecessary in uncomplicated enterococcal BSIs, while emphasizing the need for confirmation in a randomized clinical trial. Our findings, therefore, within a dedicated enterococcal cohort, provide supportive evidence that a randomized trial comparing short versus long treatment is safe and ethically justified. The ongoing INTENSE trial (7 vs 14 days for uncomplicated enterococcal BSI, with early oral switch; clinicaltrials.gov NCT05394298) will provide randomized evidence answering this question [40].

Only 870 of 3450 patients (25%) met our eligibility criteria for uncomplicated monomicrobial *E. faecalis* or *E. faecium* bacteremia, and 485 (14% of the original cohort) fulfilled the predefined treatment duration strategies for the target trial emulation. This cohort reflects the deliberate exclusion of polymicrobial bacteremia (654/3450, 19%) and clearly complicated infections (601/3450, 17%) patients, thereby focusing on patients most likely to represent “uncomplicated” disease. These proportions are consistent with published epidemiologic data demonstrating that a substantial proportion of enterococcal bloodstream infections are complicated or polymicrobial [4, 41–43]. Importantly, these exclusions were driven by clinical characteristics rather than retrospective study design constraints and therefore represent categories that a prospective randomized trial would also be expected to exclude under similar definitions. Yet, our cohort reflects a clinically meaningful real-world population in whom uncomplicated enterococcal BSI is often encountered: older, multimorbid adults with long hospitalizations and substantial 90-day readmissions and mortality, presented with short, clinically mild BSI episodes (brief bacteremia, low ICU care use, and in nearly three-quarters of cases only 1 or 2 positive blood cultures were documented). These features make shorter courses reasonable from a stewardship and safety standpoints when “uncomplicated” BSI is rigorously verified in this population. With respect to external validity, our findings reflect routine clinical practice across a large and demographically diverse healthcare network. The inclusion of older, multimorbid patients commonly encountered in real-world settings supports generalizability to similar high-income healthcare systems with comparable patient profiles and antimicrobial stewardship practices. Nevertheless, caution is warranted when extrapolating our findings to healthcare systems with substantially different resistance epidemiology or care structures, and to higher-risk populations, including patients with cardiac devices, for whom subgroup analyses were limited in size, and patients with VRE BSI, who were underrepresented.

Approximately one-fifth of the cohort had prosthetic valves or CIEDs, a group traditionally considered at higher risk for IE and relapse. Short-course therapy did not appear to result in higher 30-day mortality among these patients. In an exploratory analysis of 90-day microbiological recurrence within this subgroup, rates were similar between treatment strategies (9.6% vs 9.1%; aRR 1.06; 95% CI, 0.50 to 2.08). These recurrence rates are consistent with prior literature; Dahl et al reported recurrence rates of 4% among patients with enterococcal BSI without IE and 7% among those with IE despite adequate therapy [41]. Interpretation in our cohort is limited as patients treated >21 days were excluded, likely enriching for lower-severity device cases. Moreover, the cardiac device subgroup was modest in size with wide CIs. Thus, while short-course therapy appeared reasonable in carefully selected uncomplicated device carriers, adequately powered prospective studies, ideally with prespecified stratification for cardiac devices, are needed to confirm optimal duration in this high-risk group.

Our findings also highlight the importance of addressing selection and immortal time biases in observational studies, in this case by using a target trial emulation framework. In crude unadjusted analyses, shorter therapy appeared to have substantially higher mortality (Supplementary Figures 2 and 3). However, this was largely driven by early deaths among patients who, by definition, could not complete longer courses. Although excluding early deaths markedly attenuated the crude mortality difference between groups, doing so artificially removes a clinically relevant subgroup and therefore does not accurately reflect the true mortality risk in the population. By contrast, IPCW-adjusted analyses designed to account for informative censoring and immortal time biases estimated nearly identical risks, providing estimates that more closely approximate the effect of treatment duration on 30-day mortality. This comparison illustrates how naïve analyses can overstate the apparent benefit of long therapy, while target trial emulation with IPCW yields estimates more closely approximating causal contrasts.

Key strengths of our study include the large multicenter design, the use of comprehensive hospital-community linked EHRs, and application of a target trial emulation framework. The inclusion of diverse hospital types within Israel's largest healthcare organization enhances generalizability and modeling hospitals as a RE helped address potential center-level variability. Nevertheless, several limitations deserve consideration. First, reliance on ICD codes for case identification and classification may have introduced inaccuracies, as coding practices are not standardized between individual coders and can be nonspecific. Although an infectious diseases specialist reviewed diagnostic codes to improve classification, misclassification remains possible. Second, the relatively low use of echocardiography and positron emission tomography/computed tomography (PET/CT) may have contributed to under-recognition of IE or deep-seated infection; however, recurrence remained low across groups, suggesting few clinically significant cases missed. Third, as an observational study, residual confounding cannot be excluded. Treatment duration decisions may have reflected unmeasured clinical factors such as physician judgment or subtle patient characteristics not captured in our available data. Fourth, estimation of postdischarge antibiotic use was less precise than in-hospital administration records. Although Clalit's integrated hospital-community EHR system provides comprehensive linkage of inpatient and outpatient pharmacy data, actual adherence may not be fully captured. However, as most patients had long lengths of stay, we assume the majority of patients completed their antibiotic treatment course in-hospital and therefore, this limitation is unlikely to have a major impact on the results. Fifth, subgroup analyses for uncommon exposures such as VRE infections were underpowered, limiting interpretation of this subpopulation. Finally, although follow-up data were comprehensive within CHS, patients seeking care outside the network were not captured. This limitation is likely modest as Clalit covers over half of Israel's population and most patients typically return to the same hospital for ongoing care.

Randomized controlled trials remain the gold standard to definitively establish optimal duration. Our findings add to the growing body of supportive observational evidence, but a dedicated randomized trial is needed to confirm the safety of short-course therapy for enterococcal BSI and to guide practice. The ongoing INTENSE trial will be particularly informative, and further pragmatic international collaborative trials should aim to validate these findings across diverse patient populations, including populations underrepresented in our study such as those with *E. faecium* or VRE. Future research should also evaluate the role of oral switch therapy and assess patient-level predictors of recurrences, particularly among high-risk patients with cardiac devices or previous IE.

## CONCLUSION

In summary, to our knowledge, this multicenter target trial emulation is the largest study to date focused specifically on uncomplicated *E. faecalis* and *E. faecium* BSI. Our finding that short-course antibiotic therapy yields outcomes comparable to long therapy aligns with recent observational studies and supports the rationale for conducting large, definitive randomized trials. In the meantime, shorter treatment durations may reasonably be considered in carefully selected patients.

## Supplementary Material

ciag387_Supplementary_Data

## Supplementary Data

Supplementary materials are available at *Clinical Infectious Diseases* online. Consisting of data provided by the authors to benefit the reader, the posted materials are not copyedited and are the sole responsibility of the authors, so questions or comments should be addressed to the corresponding author.
