# Supplementary material for: Antibiotic Treatment Duration for Uncomplicated Monomicrobial Enterococcal Bloodstream Infection: A Multicenter Target Trial Emulation
Source: Clin Infect Dis. 2026 Jul 3;83(4):e718–29. doi: 10.1093/cid/ciag387 (PMC13715991; doi:10.1093/cid/ciag387)
Supplement: Supplementary file 1 [file ciag387_supplementary_data.docx]

**Supplementary materials:**

**Antibiotic Treatment Duration for Uncomplicated Monomicrobial Enterococcal Bloodstream Infection: A Multicenter Target Trial Emulation**

*List of abbreviations
Table S1. Participating hospitals
Clinical definitions* *and assumptions
Table S2. Classification of empiric, combination and definitive anti-enterococcal antibiotic therapy
Methods: Emulation framework: cloning, censoring and weighting
Table S3. Fixed effects for IPCW
Table S4. Classification of source of infection based on ICD discharge diagnosis codes
Table S5. Post-hoc IPCW-weighted estimates of absolute risk and treatment effects for cardiac-device
Table S6. Baseline patient characteristics (n=870, ≤8 days vs 8< days treatment duration)
Table S7. Sensitivity analysis of ≤8 days vs 8< days treatment duration on primary and secondary binary outcomes
Figure S1. Love plot - primary analysis
Figure S2. Crude vs adjusted 30-day mortality estimates
Figure S3. Kaplan–Meier curves for 30-day survival by treatment duration
Figure S4. Love plot - 8-day treatment duration cut-off*

**List of Abbreviations**

BSI - Bloodstream infection

CHS - Clalit Health Services

CDC - Centers for disease control and prevention

NHSN - National healthcare safety network

IPCW - Inverse probability of censoring weights

CI - Confidence intervals

IE - Infective endocarditis

VRE - Vancomycin-resistant enterococci

EHR - Electronic health record

ICD - International Classification of Diseases

SMD - Standardized mean differences

CIED - Cardiac implantable electronic devices

aRD - Adjusted risk difference

aRR - Adjusted risk ratio

PET/CT - Positron emission tomography/computed tomography

Data were obtained from eight hospitals across CHS, Israel:

| Table S1. Participating hospitals | | |
| --- | --- | --- |
| Hospital | **Type/level of care** | **Admission beds** |
| Soroka medical center, Beer Sheva | Tertiary | 1,191 |
| Beilinson campus - Rabin medical center, Petah Tikva | Tertiary | 858 |
| Meir medical center, Kfar Saba | Secondary | 797 |
| Emek medical center, Afula | Secondary | 670 |
| Kaplan medical center, Rehovot | Secondary | 581 |
| Carmel medical center, Haifa | Secondary | 489 |
| Golda Hasharon campus - Rabin medical center, Petah Tikva | Secondary | 241 |
| Yoseftal medical center, Eilat | Peripheral regional | 71 |
| Total 4,898 | | |

**Clinical definitions and assumptions:**

**Uncomplicated BSI -** Defined as a bloodstream infection without evidence of IE, abscess, bone or joint, device-related, or other deep-seated focus. Classification of uncomplicated versus complicated infection was determined by an infectious diseases specialist based on review of discharge ICD codes (Table S4).

**Monomicrobial enterococcal BSI** - Defined as a bloodstream infection caused solely by E. faecalis or E. faecium. Patients with growth of another pathogens in the same blood culture, or within 48 hours before or after the index culture were excluded. Cases in which bacterial isolates were classified as contaminants were not excluded (based on the centers for disease control and prevention [CDC]’s national healthcare safety network [NHSN] organisms list. Available from: https://www.cdc.gov/nhsn/pdfs/validation/2019/2019-NHSN-Organisms-List-Validation.xlsx).

**Exposure definition -** The exposure of interest was the duration of appropriate antibiotic therapy, defined as receipt of an agent active against the isolated *Enterococcus* species based on in-vitro susceptibility testing (supplementary Table S2). This included both empiric and definitive therapy, irrespective of route of administration (intravenous or oral). Treatment strategies were defined as short (7± 2 days) vsersus long (14± 2 days) courses of total appropriate anti-enterococcal therapy, counted from the collection date of the index culture, provided that active therapy was initiated within 72 hours of the index culture collection. Duration encompassed both inpatient therapy and post-discharge prescriptions dispensed within 96 hours after discharge through community clinics. Short gaps (≤ 72 hours) between inpatient antibiotic regimen changes were permitted and counted toward total treatment duration, allowing accurate capture of intermittently dosed agents such as vancomycin in patients with renal impairment.

**Bacteremia duration** - Defined as the number of days from the first positive blood culture to the last positive culture with the same E*nterococcus* species. Positive cultures obtained more than 21 days after the index culture and completion of antibiotic therapy were considered a recurrence.

**Microbiological recurrence** - Defined as a positive blood culture yielding the same *Enterococcus* species with identical susceptibility profile, obtained more than 21 days after the index culture and completion of antibiotic therapy

**Hospital acquired BSI** - Defined as a positive index blood culture collected after 72 hours of admission.

**Persistent bacteremia** - Defined as a positive blood culture obtained 48 hours or more after initiation of appropriate antibiotic therapy.

**Mechanical ventilation** - Defined as ICD code indicating invasive ventilation administrated within 24 hours before or after collection of index positive culture.
**Vasopressor use -** Defined as the administration of any vasoactive agent (epinephrine, norepinephrine, vasopressin, dopamine or dobutamine) within 24 hours before or after the index culture collection.

**Serum creatinine** (mg/dL) - Defined as the maximum recorded value within 24 hours before or after the index culture collection.

**Combination therapy** – Defined as the administration of ceftriaxone or gentamycin in combination with appropriate anti-enterococcal antibiotic after the collection of the index positive blood culture.

**Source of BSI/site of infection –**Defined using ICD discharge codes reviewed by infectious diseases specialist and categorized as urinary tract, intra-abdominal, vascular catheter-related, gynecological, skin/soft tissue or unspecified (Table S4).

**Treatment duration -** Counted from the collection date of the index culture. Duration encompassed both inpatient therapy and post-discharge prescriptions dispensed within 96 hours after discharge through community clinics. Short gaps (≤ 72 hours) between inpatient antibiotic regimen changes were permitted and accounted toward total treatment duration. Both empiric and definitive therapy active against the isolated species were considered appropriate, whether given intravenously or orally (supplementary Table S2 lists the empiric and definitive agents defined as active anti-enterococcal antibiotics).

***Clostridioides difficile* infection** – Defined as a positive glutamate dehydrogenase (GDH) antigen and toxin enzyme immunoassay (EIA), or in the case of discrepant results, a positive nucleic acid amplification test (NAAT) for the toxin in stool during the 90-day follow-up after the positive index culture.

| Table S2. Classification of empiric, definitive and combination anti-enterococcal antibiotic therapy | | |
| --- | --- | --- |
| Appropriate definitive anti-enterococcal antibiotic agents | | |
| Agent name | **Route of administration** | **ATC code** |
| Penicillin | Intravenous | J01CE01 |
| Ampicillin | Intravenous | J01CA01 |
| Amoxicillin | Oral | J01CA04 |
| Vancomycin^*^ | Intravenous | J01XA01 |
| Linezolid^**^ | Intravenous or oral | J01XX08 |
| Daptomycin^**^ | Intravenous | J01XX09 |
| Appropriate empiric treatment agents with enterococcal coverage | | |
| Agent name | **Route of administration** | **ATC code** |
| Piperacillin | Intravenous | J01CA12 |
| Amoxicillin clavulanic acid | Intravenous OR oral | J01CR02 |
| Piperacillin tazobactam | Intravenous | J01CR05 |
| Imipenem | Intravenous | J01DH51 |
| Tigecycline^**^ | Intravenous | J01AA12 |
| Ceftaroline^**^ | Intravenous | J01DI02 |
| Fosfomycin^**^ | Intravenous | J01XX01 |
| Combination therapy agents | | |
| Agent name | **Route of administration** | **ATC code** |
| Ceftriaxone | Intravenous | J01DD04 |
| Gentamycin | Intravenous | J01GB03 |
| *Appropriate for ampicillin resistant *enterococci* isolates **Appropriate for ampicillin and vancomycin resistant *enterococci* isolates | | |

**Methods: Emulation framework: cloning, censoring and weighting**

We emulated randomization using the cloning, censoring and inverse probability of censoring weights (IPCW) framework, which has been previously described for target trial emulation of trials comparing treatment duration. Each eligible patient was cloned to both strategies at time zero and censored upon deviating from the assigned duration window (Table 1 and Figure 1). To account for informative (dependent) censoring, stabilized IPCW were estimated separately within each treatment strategy arm using Cox proportional hazards models, in which censoring (deviation from the assigned strategy) was treated as the event. These models estimated the probability of remaining uncensored over time. Stabilized weights were calculated as the inverse of this estimated probability and incorporated into weighted regression models to estimate marginal treatment effects. Hospital site was included as a random effect to account for within-hospital clustering. Baseline covariates were selected a priori based on clinical reasoning as factors potentially influencing a clinician’s decision to continue therapy for a shorter or longer duration. These included demographic characteristics, comorbidity burden, admission characteristics, baseline infection-related variables and markers of illness severity at presentation, as well as presence of a cardiac device (A complete list is provided in supplementary Table S3). Weights were truncated at the 99^th^ percentile to minimize the influence of extreme values. Follow-up began at time zero (day 4 from index positive culture) and continued until death or 90 days after the index blood culture collection, whichever occurred first.

| Table S3. Fixed effects for IPCW | | |
| --- | --- | --- |
| Fixed factors | **Comments** | **Type** |
| Age | In years | Continuous |
| Sex | Female/Male | Categorical |
| Admission department | a) Internal medicine b) Surgery c) Gynecology d) Intensive care units (ICUs) | Categorical |
| Cardiac device | Prosthetic heart valve or pacemaker | Binary |
| Charlson comorbidity index minus age | 19 parameters, 0-24 points. CCI scores of:   a) 1–2: mild b) 3–4: moderate c) ≥5: severe | Categorical, Ordinal |
| Source of infection | a) Urinary b) Intra-abdominal c) Pelvic infections d) Central and peripheral line infections e) Skin infections f) Unspecified | Categorical |
| Hospital onset bacteremia | Positive index culture after 72 hours of hospitalization | Binary |
| Enterococcal species | a) *Faecalis* b) *Faecium* | Binary |
| Resistance patterns | a) Sensitive b) Vancomycin resistant | Categorical |
| Vasopressors use | Yes/no | Binary |
| Creatinine level | mg/dL | Continuous |
| Year of admission | 2013-2016, 2017-2020, 2021-2023 | Categorical |

| Table S4. Classification of source of infection based on ICD discharge diagnosis codes | | |
| --- | --- | --- |
| *Uncomplicated BSI cases* |  |  |
| Source of infection category | **ICD code** | **ATD code** |
| *Urine tract infections* | | |
| URINARY TRACT INFECTION, SITE NOT SPECIFIED | N39.0 | ATD_5990 |
| URINARY OBSTRUCTION, UNSPECIFIED | N13.9 | ATD_5996 |
| URINARY COMPLICATIONS, NOT ELSEWHERE CLASSIFIED | N99.89 | ATD_9975 |
| FITTING AND ADJUSTMENT OF URINARY DEVICES | Z46.6 | ATD_V536 |
| ATTENTION TO OTHER ARTIFICIAL OPENING OF URINARY TRACT | Z43.6 | INP_V556 |
| ANTEPARTUM INFECTIONS OF GENITOURINARY TRACT | B99.9 | ATD_64663 |
| COMPLICATIONS DUE TO GENITOURINARY DEVICE,IMPLANT AND GRAFT | T83.9 | ATD_99676 |
| OTHER MECHAN.COMPLIC.OF GENITOURINARY DEVICE,IMPLANT,GRAFT | T83.9 ; T85.9 | ATD_99639 |
| HYPERPLASIA OF PROSTATE, UNSPECIFIED, WITH URINARY OBSTRUCTION | N40 | ATD_60091 |
| CALCULUS OF LOWER URINARY TRACT, UNSPECIFIED | N21.9 | ATD_5949 |
| POSTPARTUM INFECTIONS OF GENITOURINARY TRACT | O86.4 | ATD_64664 |
| INFECTION/INFLAMM. DUE TO INDWELLING URINARY CATHETER | N39.0 ; T85.79 ; Y82.8 | ATD_99664 |
| OTHER LOWER URINARY TRACT CALCULUS | N21.8 | ATD_5948 |
| INFECTIONS OF GENITOURINARY TRACT IN PREGNANCY, WITH DELIVERY | O23.90 | ATD_64661 |
| ENCOUNTER FOR AFTERCARE FOLLOW.SURG.OF THE GENITOURINARY SYST.NEC | Z98.890 | ATD_V5876 |
| URINARY CALCULUS, UNSPECIFIED | N20.9 | ATD_5929 |
| CARCINOMA IN SITU OF OTHER AND UNSPECIFIED URINARY ORGANS | D09.1 | ATD_2339 |
| INFECT.OF GENITOURINARY TR.IN PREGN.,WITH DELIV.,WITH POSTP.COMPL. | O98.919 | ATD_64662 |
| SYMPTOMS INVOLVING URINARY SYSTEM | N39 | ATD_788 |
| PYELONEPHRITIS ACUTE | N10 | AMB_5901 |
| PYELONEPHRITIS, UNSPECIFIED | N12 | ATD_59080 |
| PYELONEPHRITIS OR PYONEPHROSIS,NOT SPEC.AS ACUTE OR CHR. | N13.6 | ATD_5908 |
| CHRON.PYELONEPHRITIS WITHOUT LESION OF RENAL MEDULL.NECROSIS | N11.9 | ATD_59000 |
| *Intra-abdominal infections* | | |
| CHOLANGITIS | K83.0 | ATD_5761 |
| CALCUL.OF GALLBLADDER WITH AC.CHOLECYSTITIS,WITHOUT OBSTRUCTION | K80.00 | ATD_57400 |
| ACUTE CHOLECYSTITIS | K81.0 | ATD_5750 |
| CALC.OF GALLBLADDER WITH ACUTE CHOLECYSTITIS,WITH OBSTRUCTION | K81.0 ; K82.0 | ATD_57401 |
| CHOLECYSTITIS, UNSP. | K81.9 | ATD_57510 |
| CALCULUS OF BILE DUCT WITHOUT CHOLECYSTITIS,WITHOUT OBSTR. | K80 | ATD_57450 |
| CALCUL.OF GALLBLADDER WITHOUT CHOLECYSTITIS,WITHOUT OBSTR. | K80.20 | ATD_57420 |
| CALCULUS:GALLBLADDER & BILE DUCT+AC. CHOLECYSTITIS,+ OBSTRUCTION | K80.62 ; K80.70 | ATD_57461 |
| CHOLECYSTITIS, ACUTE & CHRONIC | K81.2 | ATD_57512 |
| CALCULUS:GALLBLADDER & BILE DUCT WITHOUT CHOLECYSTITIS OR OBSTRUC | K80.70 | ATD_57490 |
| CALCULUS OF BILE DUCT WITH OTHER CHOLECYSTITIS,WITH OBSTR. | K80.40 | ATD_57441 |
| CHOLECYSTITIS, CHRONIC | K81.1 | ATD_57511 |
| RETAINED CHOLELITHIASIS FOLLOWING CHOLECYSTECTOMY | K91.86 | ATD_99741 |
| CALCULUS OF BILE DUCT WITH ACUTE CHOLECYSTITIS,WITH OBSTR. | K80.43 | ATD_57431 |
| CALCUL.OF GALLBLADDER WITH OTHER CHOLECYSTITIS,WITH OBSTR. | K80.19 | ATD_57411 |
| CALCULUS OF GALLBLADDER WITH ACUTE CHOLECYSTITIS | K80.0 | ATD_5740 |
| CALCULUS OF BILE DUCT WITH OTHER CHOLECYSTITIS,WITHOUT OBSTR. | K80.40 ; K80.50 | ATD_57440 |
| CALCUL.OF BILE DUCT WITH ACUTE CHOLECYSTITIS WITHOUT OBSTR. | K80.42 | ATD_57430 |
| OTHER CHOLECYSTITIS | K81 | ATD_5751 |
| CALCULUS:GALLBLADDER & BILE DUCT+OTHER CHOLECYSTITS,+ OBSTRUCTION | K80.6 ; K80.70 | ATD_57471 |
| CALCULUS:GALLBLADDER & BILE DUCT+AC.CHOLECYSTITIS,WITHOUT OBSTRUC | K80.00 ; K80.42 ; K80.62 ; K80.70 | ATD_57460 |
| *Vascular catheter-related infections* |  |  |
| INFECTION DUE TO CENTRAL VENOUS CATHETER | T80.21 | ATD_99931 |
| BLOODSTREAM INFECTION DUE TO CENTRAL VENOUS CATHETER | T80.211 | ATD_99932 |
| LOCAL INFECTION DUE TO CENTRAL VENOUS CATHETER | T80.212 | ATD_99933 |
| BLOODSTREAM INFECTION DUE TO CENTRAL  VENOUS CATHETER | T80.211 | ATD_99932 |
| LOCAL INFECTION DUE TO CENTRAL VENOUS CATHETER | T80.212 | ATD_99933 |
| INFECTION & INFLAMM. REACTION DUE TO  PERITONEAL DIALYSIS CATHETER | T81.9 ; T82.7 | ATD_99668 |
| MECHANICAL COMPLICATIONS DUE TO PERITONEAL  DIALYSIS CATHETER | T85.691 | ATD_99656 |
| INFECTION & INFLAMM. REACTION DUE TO  PERITONEAL DIALYSIS CATHETER | T81.9 ; T82.7 | ATD_99668 |
| MECHANICAL COMPLICATIONS DUE TO PERITONEAL  DIALYSIS CATHETER | T85.691 | ATD_99656 |
| *Gynecological infections* |  |  |
| UNSPECIFIED TYPE MATERNAL PYREXIA DURING LABOR DELIVERED | O75.2 | ATD_65921 |
| INFECTION OF AMNIOTIC CAVITY DELIVERED | O41.1090 | ATD_65841 |
| UNSPECIFIED TYPE MATERNAL PYREXIA DURING LABOR DELIVERED | O75.2 | ATD_65921 |
| COMPLICATION OF OBSTETRICAL SURG.WOUNDSPOSTPART.CONDIT./COMPL. | O90.89 | ATD_67434 |
| RETAINED PLACENTA WITHOUT HEMORRHAGEPOSTPARTUM COMPLICATION | O73.0 | ARD_66704 |
| POST TERM PREGNANCY ANTEPARTUM | O48.0 | ATD_64513 |
| PUERPERAL ENDOMETRITIS POSTPARTUM CONDITION OR COMPLICATION | O86.12 | ATD_67014 |
| *Skin/soft tissue infections* |  |  |
| PRESSURE ULCER STAGE III | L89.93 | ATD_70723 |
| PRESSURE ULCER STAGE II | L89.92 | ATD_70722 |
| CELLULITIS AND ABSCESS OF LEG EXCEPT FOOT | L02.419; L03.119; L08.0 | ATD_6826 |
| DECUBITUS ULCER HEEL | L89.609 | ATD_70707 |
| PRESSURE ULCER STAGE IV | L89.94 | ATD_70724 |
| PRESSURE ULCER LOWER BACK | L89.109 | ATD_70703 |
| CELLULITIS AND ABSCESS OF HAND EXCEPT FINGERS AND THUMB | L02.519; L03.119 | ATD_6824 |
| DECUBITUS ULCER OTHER SITE | L98.499 | ATD_70709 |
| CELLULITIS AND ABSCESS OF OTHER SPECIFIED SITES | L02.91; L03.90 | ATD_6828 |
| *Complicated BSI cases* |  |  |
| Source of infection category | **ICD code** | **ATD code** |
| *Endocarditis* |  |  |
| ENDOCARDITIS BACTERIAL SUBACUTE | B96.89 ; I33.0 | AMB_4210 |
| ACUTE + SUBACUTE INFEC.ENDOCARDITIS IN DIS.CLASS.ELSEWHERE | B99.9 ; I33.9 | ATD_4211 |
| ACUTE AND SUBACUTE ENDOCARDITIS | I33 | ATD_421 |
| ACUTE AND SUBACUTE BACTERIAL ENDOCARDITIS | I33.0 | ATD_4210 |
| ACUTE ENDOCARDITIS, UNSPECIFIED | I33.9 | ATD_4219 |
| ENDOCARDITIS, VALVE UNSPECIFIED, UNSPECIFIED CAUSE | I38 | ATD_42490 |
| ENDOCARDITIS IN DISEASES CLASSIFIED ELSEWHERE | I39 | ATD_42491 |
| ENDOCARDITIS BACTERIAL SUBACUTE | B96.89 ; I33.0 | AMB_4210 |
| ACUTE + SUBACUTE INFEC.ENDOCARDITIS IN DIS.CLASS.ELSEWHERE | B99.9 ; I33.9 | ATD_4211 |
| ACUTE AND SUBACUTE ENDOCARDITIS | I33 | ATD_421 |
| ACUTE AND SUBACUTE BACTERIAL ENDOCARDITIS | I33.0 | ATD_4210 |
| ACUTE ENDOCARDITIS, UNSPECIFIED | I33.9 | ATD_4219 |
| ENDOCARDITIS, VALVE UNSPECIFIED, UNSPECIFIED CAUSE | I38 | ATD_42490 |
| ENDOCARDITIS IN DISEASES CLASSIFIED ELSEWHERE | I39 | ATD_42491 |
| INFECTION/INFLAMM. DUE TO CARDIAC DEVICE, IMPLANT AND GRAFT | Z94.1 | ATD_99661 |
| *Deep-seated abscesses* |  |  |
| ABSCESS OF LIVER | K75.0 | ATD_5720 |
| ABSCESS OF INTESTINE | K63.0 | ATD_5695 |
| INTRASPINAL ABSCESS | G06.1 | ATD_3241 |
| PERITONEAL ABSCESS | K65.1 | ATD_56722 |
| INTRACRANIAL AND INTRASPINAL ABSCESS OF UNSPECIFIED SITE | G06.2 | ATD_3249 |
| OTHER ORCHITIS,EPIDIDYMITIS,EPIDIDYMO-ORCHIT. WITHOUT ABSCESS | N45.1 ; N45.3 | ATD_60499 |
| INTRACRANIAL ABSCESS | G06.0 | ATD_3240 |
| ABSCESS OF ANAL AND RECTAL REGIONS | K61 | ATD_566 |
| PALMAR SPACE ABSCESS | L02.519 | AMB_6824 |
| RENAL AND PERINEPHRIC ABSCESS | N15.1 | ATD_5902 |
| PSOAS MUSCLE ABSCESS | K68.12 | ATD_56731 |
| ABSCESS OF PROSTATE | N41.2 | ATD_6012 |
| ABSCESS PROSTATE | N41.2 | AMB_6012 |
| LIVER ABSCESS AND SEQUELAE OF CHRONIC LIVER DISEASE | K75.0 | ATD_572 |
| ABSCESS INGUINAL | L02.214 | AMB_6822 |
| ABSCESS OF LUNG | J85.0 | ATD_5130 |
| RECURRENT ABSCESS OF BREAST | N61.1 | AMB_6110 |
| URETHRAL ABSCESS | N34.0 | ATD_5970 |
| LATE EFFECTS OF INTRACRANIAL ABSCESS OR PYOGENIC INFECTION | G09 | ATD_326 |
| ORCHITIS, EPIDIDYMITIS, AND EPIDIDYMO-ORCHITIS, WITH ABSCESS | N45.4 | ATD_6040 |
| ABSCESS OF EYELID | H00.03 | ATD_37313 |
| ACUTE APPENDICITIS WITH PERITONEAL ABSCESS | K35.3 | ATD_5401 |
| PERIANAL ABSCESS | K61.0 | AMB_566 |
| AMEBIC LIVER ABSCESS | A06.4 | INP_0063 |
| *Bone and joint infections* |  |  |
| UNSPECIFIED OSTEOMYELITIS INVOLVING FOREARM | M86.9 | ATD_73023 |
| ACUTE OSTEOMYELITIS INVOLVING LOWER LEG | M86.169 | ATD_73006 |
| CHRONIC OSTEOMYELITIS INVOLVING OTHER SPECIFIED SITES | M86.60 | ATD_73018 |
| ACUTE OSTEOMYELITIS, SITE UNSPECIFIED | M86.10 | ATD_73000 |
| CHRONIC OSTEOMYELITIS | M86.6 | ATD_7301 |
| CHRONIC OSTEOMYELITIS INVOLVING MULTIPLE SITES | M86.69 | ATD_73019 |
| ACUTE OSTEOMYELITIS INVOLVING MULTIPLE SITES | M86.19 | ATD_73009 |
| CHRONIC OSTEOMYELITIS INVOLVING ANKLE AND FOOT | M86.679 | ATD_73017 |
| CHRONIC OSTEOMYELITIS INVOLVING HAND | M86.649 | ATD_73014 |
| ACUTE OSTEOMYELITIS INVOLVING HAND | M86.149 | ATD_73004 |
| OSTEOMYELITIS, PERIOSTITIS, AND OTHER INFECTIONS INVOLVING BONE | M86 | ATD_730 |
| ACUTE OSTEOMYELITIS INVOLVING PELVIC REGION AND THIGH | M86.159 ; M86.18 | ATD_73005 |
| ACUTE OSTEOMYELITIS | M86.1 | ATD_7300 |
| CHRONIC OSTEOMYELITIS INVOLVING PELVIC REGION AND THIGH | M86.659 ; M86.68 | ATD_73015 |
| CHRONIC OSTEOMYELITIS INVOLVING LOWER LEG | M86.669 | ATD_73016 |
| UNSPECIFIED INFECTIVE ARTHRITIS INVOLVING LOWER LEG (INCL.KNEE) | M00.9 | ATD_71196 |
| PYOGENIC ARTHRITIS INVOLVING SHOULDER REGION | M00.9 ; M13.819 | ATD_71101 |
| PYOGENIC ARTHRITIS INVOLVING HAND | M00.849 | ATD_71104 |
| *Non-cardiac device related infections* | | |
| MECHANICAL COMPLIC. DUE TO IMPLANT & INTERNAL DEVICE N.E.C._- | T85.698 ; T85.9 | ATD_99659 |
| POSTSURGICAL PRESENCE OF CEREBROSPINAL FLUID DRAINAGE DEVICE | Z98.890 | ATD_V452 |
| COMPLICATIONS DUE TO OTHER INTER.PROSTHE. DEVICE,IMPLANT & GRAFT | T85.9 | ATD_99679 |
| COMPLICATIONS DUE TO GENITOURINARY DEVICE,IMPLANT AND GRAFT | T83.9 | ATD_99676 |
| INFECTION/INFLAMM.DUE TO OTHER VASCULAR DEVICE,IMPLANT AND GRAFT | T82.7 ; Y71.2 | ATD_99662 |
| UNSPEC.MECHANI.COMPL.OF INTERNAL ORTHOPEDIC DEVICE,IMPLANT,GRAFT | T84.9 ; T85.9 | ATD_99640 |
| OTHER MECHAN.COMPLIC.OF GENITOURINARY DEVICE,IMPLANT,GRAFT | T83.9 ; T85.9 | ATD_99639 |
| COMPLICATIONS DUE TO RENAL DIALYSIS DEVICE,IMPLANT AND GRAFT | T80.90 | ATD_99673 |
| INFECTION/INFLAMM.DUE TO OTHER INT.PROSTHET.DEVICE,IMPLANT,GRAFT | T85.79 | ATD_99669 |
| INFECTION/INFLAMM.DUE TO OTHER GENITOURINARY DEVICE,IMPLANT,GRAFT | B99.9 | ATD_99665 |
| MECHANICAL COMPLICATION,OTHER VASCULAR DEVICE,IMPLANT,GRAFT | T82.398 | ATD_9961 |
| COMPLICATIONS DUE TO OTHER VASCULAR DEVICE,IMPLANT AND GRAFT | T82.9 | ATD_99674 |
| ENCOUNTER FOR REMOVAL OF INTERNAL FIXATION DEVICE | Z47.2 | ATD_V5401 |
| OTHER MECHAN.COMPLIC.OF OTHER INTER.ORTHOPED.DEVICE,IMPLANT,GRAFT | T84.9 | ATD_99649 |
| FITTING AND ADJUSTMENT OF ORTHOPEDIC DEVICES | Z46.89 | ATD_V537 |
| INFECTION/INFLAMM.DUE TO NERVOUS SYSTEM DEVICE,IMPLANT,AND GRAFT | B99.9 ; G98.8 | ATD_99663 |
| Note: Cases were classified as “unspecified source” when discharge diagnoses did not include any ICD code corresponding to a defined infection source | | |

| Table S5. Post-hoc IPCW-weighted estimates of absolute risk and treatment effects for cardiac-device subgroup | | | | | | |
| --- | --- | --- | --- | --- | --- | --- |
|  | **Absolute risk  Short treatment 7±2 days** | **Absolute risk  Long treatment 14±2 days** | | **Risk Difference  (95% CI)** | | **Risk Ratio  (95% CI)** |
| **Primary outcome - 30-day all-cause mortality** |  | |  | |  |  |
| Cardiac device present | 0.203 (0.141 - 0.267) | 0.204 (0.143 - 0.266) | | -0.001 (-0.085 - 0.086) | | 0.997 (0.656 - 1.541) |
| Cardiac device absent | 0.219 (0.188 - 0.253) | 0.212 (0.178 - 0.245) | | 0.007 (-0.039 - 0.051) | | 1.034 (0.835 - 1.270) |
| **90-day all-cause mortality** |  |  | |  | |  |
| Cardiac device present | 0.354 (0.282 - 0.435) | 0.374 (0.300 - 0.466) | | -0.019 (-0.140 - 0.088) | | 0.948 (0.673 - 1.275) |
| Cardiac device absent | 0.378 (0.335 - 0.419) | 0.381 (0.341 - 0.419) | | -0.002 (-0.062 - 0.052) | | 0.994 (0.847 - 1.147) |
| **Microbiological recurrence** |  |  | |  | |  |
| Cardiac device present | 0.096 (0.052 - 0.141) | 0.091 (0.053 - 0.137) | | 0.006 (-0.061 - 0.066) | | 1.065 (0.505 - 2.081) |
| Cardiac device absent | 0.037 (0.022 - 0.051) | 0.042 (0.025 - 0.062) | | -0.005 (-0.032 - 0.017) | | 0.875 (0.449 - 1.572) |

| Table S6. Baseline patient characteristics (n=870, ≤8 days vs 8< days treatment duration) | | | | |
| --- | --- | --- | --- | --- |
| **Variable** | **Overall n=870** | **Short treatment** **≤ 8 days n=390** | **Long treatment 8< days n=480** | **SMD** |
| **Demographics** |  |  |  |  |
| Age | 73.65 (15.83) | 73.88 (16.73) | 73.46 (15.08) | 0.027 |
| Female | 337 (38.7) | 152 (39.0) | 185 (38.5) | 0.009 |
| **Comorbidities** |  |  |  |  |
| Median BMI (IQR) | 27 (23.0-31.0) | 27 (23.3-31.0) | 27 (23.0-31.0) | 0.063 |
| CCS index – age |  |  |  | 0.043 |
| Mild (1-2) | 210 (24.1) | 98 (25.1) | 112 (23.3) |  |
| Moderate (3-4) | 240 (27.6) | 107 (27.4) | 133 (27.7) |  |
| Severe (≥5) | 420 (48.3) | 185 (47.4) | 235 (49.0) |  |
| Diabetes mellitus | 440 (50.6) | 204 (52.3) | 236 (49.2) | 0.063 |
| Chronic kidney disease | 354 (40.7) | 159 (40.8) | 195 (40.6) | 0.003 |
| Congestive heart failure | 279 (32.1) | 118 (30.3) | 161 (33.5) | 0.071 |
| Ischemic heart disease | 182 (20.9) | 77 (19.7) | 105 (21.9) | 0.053 |
| Cardiac device | 145 (16.7) | 52 (13.3) | 93 (19.4) | 0.164 |
| Prosthetic heart valve | 78 (9.0) | 21 (5.4) | 57 (11.9) | 0.233 |
| CIED | 82 (9.4) | 34 (8.7) | 48 (10.0) | 0.044 |
| Cerebrovascular accident | 319 (36.7) | 135 (34.6) | 184 (38.3) | 0.077 |
| Pulmonary disease | 265 (30.5) | 115 (29.5) | 150 (31.2) | 0.038 |
| Solid malignancies | 267 (30.7) | 120 (30.8) | 147 (30.6) | 0.003 |
| Hematologic malignancies | 74 (8.5) | 30 (7.7) | 44 (9.2) | 0.053 |
| Dementia | 183 (21.0) | 86 (22.1) | 97 (20.2) | 0.045 |
| Liver diseases | 108 (12.4) | 49 (12.6) | 59 (12.3) | 0.008 |
| Peripheral vascular disease | 143 (16.4) | 53 (13.6) | 90 (18.8) | 0.141 |
| Rheumatic diseases | 61 (7.0) | 25 (6.4) | 36 (7.5) | 0.043 |
| **Hospitalization details** |  |  |  |  |
| Year of infection |  |  |  | 0.155 |
| 2013-2016 | 174 (20.0) | 68 (17.4) | 106 (22.1) |  |
| 2017-2020 | 397 (45.6) | 174 (44.6) | 223 (46.5) |  |
| 2021-2023 | 299 (34.4) | 148 (37.9) | 151 (31.5) |  |
| Hospital |  |  |  | 0.079 |
| Tertiary | 401 (46.1) | 188 (48.2) | 2 (0.4) |  |
| Secondary | 465 (53.4) | 200 (51.3) | 265 (55.2) |  |
| Regional | 4 (0.5) | 2 (0.5) | 2 (0.4) |  |
| Admission ward |  |  |  | 0.125 |
| Internal medicine | 635 (73.0) | 279 (71.5) | 356 (74.2) |  |
| Surgery | 189 (21.7) | 87 (22.3)) | 102 (21.2) |  |
| Gynecology | 26 (3.0) | 16 (4.1) | 10 (2.1) |  |
| Intensive care unit | 20 (2.3) | 8 (2.1) | 12 (2.5) |  |
| **Infection severity** |  |  |  |  |
| Mechanical ventilation | 28 (3.2) | 12 (3.1) | 16 (3.3) | 0.015 |
| Vasopressors | 79 (9.1) | 47 (12.1) | 32 (6.7) | 0.186 |
| Median creatinine (mg/dL) (IQR) | 1.22 (0.9-1.9) | 1.3 (0.9-2.1) | 1.2 (0.8-1.8) | 0.102 |
| **Bacteremia characteristics** |  |  |  |  |
| Source of Infection |  |  |  | 0.330 |
| Urinary tract | 266 (30.6) | 127 (32.6) | 139 (29.0) |  |
| Intra-abdominal | 120 (13.8) | 51 (13.1) | 69 (14.4) |  |
| Skin/soft tissue | 10 (1.1) | 6 (1.5) | 4 (0.8) |  |
| Gynecological | 9 (1.0) | 9 (2.3) | 0 (0.0) |  |
| Vascular catheter-related | 20 (2.3) | 2 (0.5) | 18 (3.8) |  |
| Unspecified | 445 (51.1) | 195 (50.0) | 250 (52.1) |  |
| Acquired bacteremia | 323 (37.1) | 150 (38.5) | 173 (36.0) | 0.050 |
| Median days from collection to result (IQR) | 0.7 (0.5-1.4) | 0.6 (0.5-1.3)) | 0.7 (0.5-1.4) | 0.168 |
| Median days of bacteremia (IQR) | 1.0 (1.0-1.0) | 1.0 (1.0-1.0) | 1.0 (1.0-2.0) | 0.249 |
| Total positive blood culture bottles ^a^ |  |  |  | 0.341 |
| 1 | 417 (47.9) | 215 (55.1) | 202 (42.1) |  |
| 2 | 222 (25.5) | 101 (25.9) | 121 (25.2) |  |
| 3-5 | 174 (20.0) | 59 (15.1) | 115 (24.0) |  |
| >5 | 57 (6.6) | 15 (3.8) | 42 (8.8) |  |
| Enterococci Species |  |  |  | 0.023 |
| *E. faecalis* | 718 (82.5) | 320 (82.1) | 398 (82.9) |  |
| *E. faecium* | 152 (17.5) | 70 (17.9) | 82 (17.1) |  |
| Ampicillin susceptibility (%) |  |  |  | 0.160 |
| Susceptible | 732 (84.1) | 331 (84.9) | 401 (83.5) |  |
| Non-susceptible | 115 (13.2) | 54 (13.8) | 61 (12.7) |  |
| Not performed or missing | 23 (2.6) | 5 (1.3) | 18 (3.8) |  |
| Vancomycin-resistant enterococci | 13 (1.5) | 10 (2.6) | 3 (0.6) | 0.155 |
| **Antibiotic treatment** |  |  |  |  |
| Appropriate empiric antibiotics | 163 (18.7) | 65 (16.7) | 98 (20.4) | 0.097 |
| Median days from culture collection to appropriate antibiotic treatment (IQR) | 1.6 (0.9-2.5) | 1.6 (0.9-2.5) | 1.6 (0.8-2.5) | 0.115 |
| Combination therapy ^b^ | 345 (39.7) | 133 (34.1) | 212 (44.2) | 0.207 |
| **Imaging** |  |  |  |  |
| Echocardiography ^c^ |  |  |  | 0.645 |
| Not performed | 417 (47.9) | 248 (63.6) | 169 (35.2) |  |
| Trans-thoracic | 394 (45.3) | 135 (34.6) | 259 (54.0) |  |
| Trans-esophageal | 59 (6.8) | 7 (1.8) | 52 (10.8) |  |
| PET/CT | 31 (3.6) | 6 (1.5) | 25 (5.2) | 0.204 |

Data are mean (SD) or n (%), except for variables explicitly reported as median (IQR)
^a^ Counts may include multiple cultures drawn on the same day.
^b^ Combination therapy defined as administration of ceftriaxone or gentamicin in conjunction with antibiotics with direct enterococcal activity.

^c^ Patients who underwent both TTE and TEE were classified to the TEE group.
SMD – Standardized Mean Difference, BMI – body mass index, CCS - Charlson Comorbidity Score (calculated without age adjustment to account for elderly-skewed cohort), CIED – cardiac implantable electronic devise, PET/CT - Positron Emission Tomography/Computed Tomography.

| Table S7. Sensitivity analysis of ≤8 days vs 8< days treatment duration on primary and secondary binary outcomes | | | | |
| --- | --- | --- | --- | --- |
| **Outcome** | **Absolute risk  Short treatment** ≤8 days | **Absolute risk  Long treatment** 8< days | **Risk Difference (95% CI)** | **Risk Ratio (95% CI)** |
| **Primary outcome** |  |  |  |  |
| 30-day all-cause mortality | 0.227 (0.199 - 0.254) | 0.255 (0.229 - 0.284) | -0.029 (-0.071 - 0.009) | 0.887 (0.740 - 1.041) |
| **Secondary outcomes** |  |  |  |  |
| 90-day all-cause mortality | 0.387 (0.354 - 0.418) | 0.406 (0.375 - 0.439) | -0.019 (-0.066 - 0.027) | 0.954 (0.845 - 1.071) |
| Microbiological recurrence | 0.049 (0.033 - 0.064) | 0.046 (0.032 - 0.062) | 0.003 (-0.018 - 0.024) | 1.067 (0.680 - 1.702) |
| *Clostridioides difficile* infection | 0.018 (0.008 - 0.028) | 0.017 (0.009 - 0.025) | 0.001 (-0.012 - 0.012) | 1.046 (0.453 - 2.121) |
| Recurrent admissions | 0.450 (0.414 - 0.480) | 0.424 (0.390 - 0.457) | 0.026 (-0.022 - 0.074) | 1.062 (0.949 - 1.185) |

**Supplementary figures:**

**Figure S1.** **Love plot - Primary analysis (n=485).** Covariate balance before and after IPCW. IPCW model achieved satisfactory covariate balance across treatment strategies, with all standardized mean differences (SMDs) reduced to < 0.1 following weighting.

**Figure S2. Crude vs adjusted 30-day mortality estimates.** This figure presents three approaches to estimating 30-day mortality among patients receiving short (7 ± 2 days) versus long (14 ± 2 days) antibiotic treatment: (a) *Crude:* unadjusted 30-day mortality by observed treatment duration; (b) *Crude with early deaths excluded*: excludes patients who died within 14 days of index culture collection. This approach superficially equalizes mortality between groups (8.1% vs 8.5%) but is misleading, as it removes patients who were never eligible to receive a long course, thereby underestimating the true mortality burden in the two groups; and (c) IPCW (TTE) = target-trial-emulation estimate using a cloning–censoring–weighting framework. The IPCW-adjusted results better reflect the outcomes under each treatment strategy after accounting for confounding by indication and immortal time bias.

*Numerators and denominators are shown for crude analyses only. IPCW estimates represent weighted risks from the target trial emulation and do not correspond to raw event counts.*

**Figure S3. Kaplan–Meier curves for crude unadjusted 30-day survival by treatment duration.** Curves and log-rank P value are crude (unadjusted) and therefore reflect both confounding by indication and immortal-time bias. The apparent advantage of longer therapy is therefore misleading, as these unadjusted estimates do not represent a causal effect. The P value was calculated using the log-rank test. *Note: Survival probabilities represent only patients alive and under observation. Because treatment duration was defined retrospectively, patients in the short-treatment group (5–7 days) could only enter the risk set once they survived long enough to complete that duration, whereas patients in the long-treatment group (12–16 days) could only enter the risk set after surviving to at least day 12. This explains why both curves appear after their respective minimum possible durations and reflects immortal-time bias rather than true differences in survival.*

**Figure S4.** **Love plot - 8-day treatment duration cut-off (n=870).** Covariate balance before and after IPCW in the 8-day treatment duration cutoff included in the sensitivity analysis. IPCW model achieved satisfactory covariate balance across treatment strategies, with all standardized mean differences (SMDs) reduced to < 0.1 following weighting.
